# Different responses of rhizosphere and non-rhizosphere soil microbial communities to consecutive *Piper nigrum* L. monoculture

**DOI:** 10.1038/srep35825

**Published:** 2016-10-24

**Authors:** Zhigang Li, Chao Zu, Can Wang, Jianfeng Yang, Huan Yu, Huasong Wu

**Affiliations:** 1 Spice and Beverage Research Institute, Chinese Academy of Tropical Agricultural Sciences & Key Laboratory of Genetic Resources Utilization of Spice and Beverage Crops, Ministry of Agriculture & Hainan Provincial Key Laboratory of Genetic Improvement and Quality Regulation for Tropical Spice and Beverage Crops, Wanning 571533, Hainan, P. R. China

## Abstract

Soil microorganisms have important influences on plant growth and health. In this study, four black pepper fields consecutively monocultured for 12, 18, 28 and 38 years were selected for investigating the effect of planting age on rhizosphere and non-rhizosphere soil microbial communities and soil physicochemical properties. The results revealed that the relative abundance of the dominant bacterial phyla in rhizosphere soil increased considerably with long-term consecutive monoculture but decreased in non-rhizosphere soil with a significant decline in *Firmicutes*. For fungi, an increasing trend over time was observed in both rhizosphere and non-rhizosphere soils, with the abundance of the pathogenic fungi *Fusarium* increasing significantly accompanied by a decrease in the bacteria *Pseudomonas* and *Bacillus* that is beneficial for black pepper. Consecutive monoculture, especially for 38 years, considerably decreased soil microbial diversity. Additionally, the rhizosphere soil pH and organic matter and available K contents decreased with increasing planting duration, though available N and P increased. All soil nutrient contents and microbial diversity indices were higher in rhizosphere soil compared to non-rhizosphere soil. The results suggest that long-term consecutive monoculture leads to variations in soil microbial community composition and physicochemical properties in both rhizosphere and non-rhizosphere soils, thus inhibiting the black pepper growth.

Black pepper (*Piper nigrum* L.), of the Piperaceae family, is a perennial woody climber plant that yields the commercial product known as “black pepper”^1^,^2^. Considered to be the “King of Spices”, black pepper has a wide range of uses, from a simple dietary constituent to compelling pharmacological benefits^3^,^4^. The plant has great economic importance in many tropical countries, and Vietnam, India, Indonesia, Brazil and China are the world’s major producers and exporters of the black pepper commodity^5^. Hainan Province, the major producer and exporter in China, cultivates 22,000 ha of *P. nigrum* and produces 3.6 × 10^7^ kg of dried peppercorns annually, comprising 90% of the pepper production in China. This constitutes an important tropical crop industry in China, with an output value of more than 15 million USD^6^.

However, consecutive monoculture problems are among the key actors that affect the productivity and quality of black pepper in China^7^,^8^. Although consecutive cropping is common in agricultural production, it can cause issues such as soil quality degradation^9^, crop yield reduction^10^, and aggravated crop pest-induced plant diseases^11^. For black pepper, long-term monoculture results in poor growth, low yield and serious disease, severely hindering the black pepper industry. There are a certain number of researches on black pepper consecutive monoculture problems, mainly focused on fruit quality^12^, soil nutrition and physicochemical properties^13^,^14^.

Slow decline/wilt, Phytophthora foot rot disease and root-knot nematodes are the major black pepper diseases. These three kinds of diseases occur and spread more frequently in long-term consecutive monocultured black pepper fields^5^,^7^. *Fusarium* is reported to be the pathogenic fungus of black pepper slow decline/wilt^5^, and *Pseudomonas* and *Bacillus* has antagonist effects against *Phy. Capsici*^15^ and root-knot nematode^16^. However, the changes of the abundance of the pathogenic fungus *Fusarium* and the antagonistic bacteria *Pseudomonas* and *Bacillus* in the black pepper soil with the planting age has not been reported.

The rhizosphere is a hot spot of microbial interactions, as exudates released by plant roots are the main food source for microorganisms and a driving force of their population density and activities^17^,^18^. Plants produce root exudates that stimulate heterotrophic growth, which leads to local competition between roots and microorganisms for inorganic nutrients^19^,^20^, and the root-associated microbial community plays an important role in the soil ecosystem, influencing many soil biochemical processes and impacting the growth and health of plants^21^. Although a large number of studies have shown that continuous cropping results in changes in the rhizosphere soil microbial structure, very few studies have involved black pepper. Conversely, non-rhizosphere soil is not (or only lightly) affected by plants roots and root exudates, with a consequent lower level of microbial activity and soil fertility. Nonetheless, this part of the soil is necessary for the stability of soil aggregates and the resistance of soil to negative phenomena (e.g., soil erosion, leaching of nutrients)^22^. There are few studies to date on the effect of continuous cropping on non-rhizosphere or bulk soil microbial community composition. Furthermore, comprehensive comparison of rhizosphere and non-rhizosphere soil microbial communities and the physicochemical property response to continuous cropping are rarely reported.

Soil physicochemical characteristics, especially soil nitrogen (N) availability and soil pH, have a large effect on the composition of soil microbial communities^23^,^24^. Our previous study reported that long-term black pepper consecutive monoculture led to a significant decline in soil pH, organic matter (OM), and enzymatic activities, resulting in a decrease in soil bacterial abundance^25^. In the present study, we focused in comparing the effect of long-term consecutive monoculture on the composition of bacterial and fungal communities as well as the physicochemical characteristics between rhizosphere and non-rhizosphere soils.

## Results

### Microbial community composition

After filtering reads according to basal quality control and singleton operational taxonomic unit (OTU) removal, 1,477,241 sequences comprising 9,665 of bacterial OTUs were obtained from 24 samples. The number of high-quality sequences per sample varied from 36,084 to 89,822. Classified sequences across all samples were affiliated with 25 bacterial phyla, and the remaining sequences were unclassified. The dominant phyla (average relative abundance [ARA] >1%) across all samples were *Acidobacteria* (23.41%), *Proteobacteria* (18.83%), *Firmicutes* (6.75%), *Chloroflexi* (6.37%), *Verrucomicrobia* (5.57%), *Actinobacteria* (3.91%), *Planctomycetes* (3.87%), *Bacteroidetes* (3.09%) and *Gemmatimonadetes* (1.09%) (Fig. S1a). In addition, *Nitrospira, Armatimonadete*s, *Cyanobacteria* and *WS3* were present in most soils but at relatively low abundance (ARA: 0.1–1%). Twelve other rarer phyla belonged to Others (ARA <0.1%).

Regarding fungi, after filtering reads as indicated above, pyrosequencing-based analysis of ITS sequences recovered 2,039,925 high-quality sequences across all samples, with 47,817–139,906 high-quality sequences per sample clustering into 3,249 OTUs. Based on 97% species similarity, 2,921 fungal OTUs from *Ascomycota* were predominantly observed, accounting for 89.92% of the total fungal sequences obtained (Fig. S1b). The other four phyla observed were *Basidiomycota* (2.52%), *Zygomycota* (0.91%), *Chytridiomycota* (0.44%) and *Glomeromycota* (0.01%); 6.21% of fungi were unclassified.

**Figure 1:**
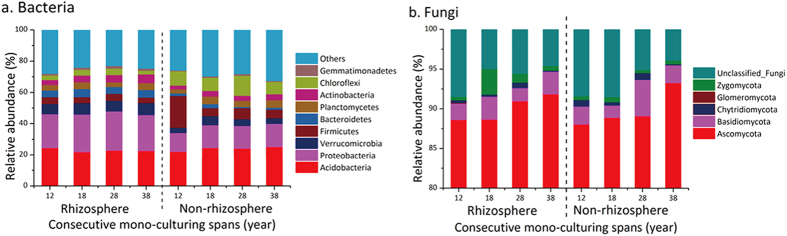
Relative abundance of main bacterial (**a**) and fungal (**b**) phylum in rhizosphere and non-rhizosphere soil at four consecutive monoculture spans.

**Figure 2:**
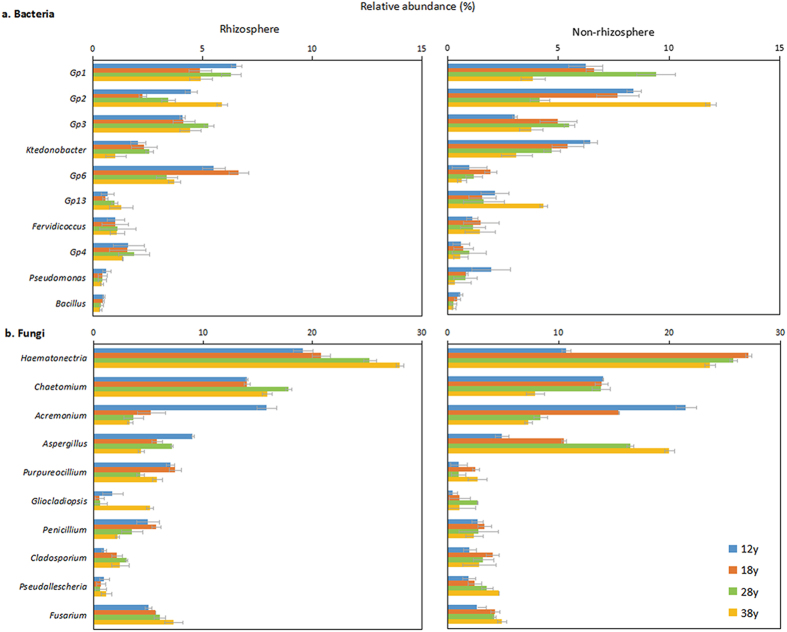
Relative abundances of the top 10 bacterial (**a**) and fungal (**b**) genera in rhizosphere and non-rhizosphere soil at four consecutive monoculture spans. The “12y”, “18y”, “28y” and “38y” refers to the four consecutive monoculture spans of 12, 18, 28 and 38 years, respectively.

The relative abundance (RA) of different phyla and genera from rhizosphere and non-rhizosphere soils were compared (Figs 1 and 2). As shown in Fig. 1a, long-term monoculture resulted in considerable increases in RA of the dominant bacteria phyla in the rhizosphere soil but decreases in the non-rhizosphere soil. In rhizosphere soil, RA of *Acidobacteria* and *Firmicutes* decreased, whereas that of *Proteobacteria, Chloroflexi, Verrucomicrobia, Actinobacteria, Planctomycetes, Bacteroidetes* and *Gemmatimonadetes* increased. In non-rhizosphere soil, RA of *Firmicutes* decreased from 20.31% (12 years) to 5.37% (38 years). In contrast, a rising trend of fungal RA was observed in both the rhizosphere and non-rhizosphere soils as planting duration increased (Fig. 1b), with the RA of *Ascomycota, Basidiomycota, Zygomycota* and *Glomeromycota* increasing in both soil types. However, greater *Zygomycota* abundance was detected in rhizosphere soil compared to non-rhizosphere soil.

At the genus level (Fig. 2), *GP4* and *GP6* genera had higher RA in rhizosphere soil than in non-rhizosphere soil, whereas the opposite trend was observed for the other eight genera. In non-rhizosphere soil, the RA of *Ktedonobacter* decreased as planting duration increased; however, the RA of other genera exhibited no obvious variation trend. For fungi, the tendencies of almost all genera in the rhizosphere soil were similar to those in the non-rhizosphere soil, except for *Aspergillus,* which decreased in the rhizosphere soil and increased in the non-rhizosphere soil.

Strikingly, the pathogenic fungus *Fusarium* increased significantly as planting duration increased in both the rhizosphere and non-rhizosphere soils. In contrast, *Pseudomonas* and *Bacillus*, beneficial for the growth of black pepper, showed a considerable decline.

### Bacterial and fungal α-diversity

The microbial community richness and diversity indices are listed in Table 1. The coverage of all samples was above 96%, indicating that the depth of the sequencing met the needs of our experiments. With regard to fungi, the Shannon index revealed a considerable decline in both rhizosphere and non-rhizosphere soils as the planting duration increased. In particular, the soil fungi diversity significantly decreased after 38 years of black pepper cultivation. For bacteria, the soil bacterial richness and diversity decreased with planting duration in both the rhizosphere and non-rhizosphere soils, even though the Chao1 and Shannon indices did not reveal a significant difference.

### Soil chemical properties and effects on bacterial or fungal taxa

As shown in Table 2, the soil OM and available K (AK) contents decreased significantly with time of monoculture in both the rhizosphere and non-rhizosphere soils. Changes in the pH value for both soils showed fluctuation with a downward trend. Furthermore, available N (AN) and available P (AP) contents increased with time in the rhizosphere soil, and the AP value of the rhizosphere soil was approximately 10 times higher than that of the non-rhizosphere soil.

**Figure 3:**
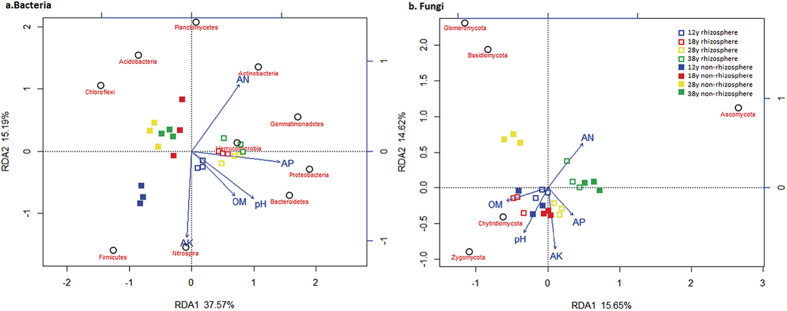
Redundancy analysis (RDA) of abundant soil bacterial (**a**) and fungal (**b**) phyla and soil physicochemical properties for individual samples from four consecutive monoculture in black pepper fields. “12y”, “18y”, “28y” and “38y” refers to the four consecutive monoculture spans of 12, 18, 28 and 38 years, respectively.

Monte Carlo tests based on soil chemical properties and the numbers of the most abundant bacterial and fungal phyla revealed that the selected soil chemical properties were significantly correlated with variations in the selected phyla (*P* = 0.01). Redundancy analysis (RDA) showed that the first and second RDA components explained 52.76% and 30.27% of the total bacterial and fungal variations, respectively (Fig. 3). For bacteria, the first component (RDA1), which explained 37.57% of the total variation, separated the rhizosphere soil samples obtained from the four field plots of different planting duration from the non-rhizosphere soil samples. As shown by their close groupings and vectors, the dominant bacterial phylum in the rhizosphere soil samples was *Proteobacteria* and was related to AP, pH, and OM and AN contents, whereas the dominant bacterial phylum in the non-rhizosphere soil was *Acidobacteria*. Regarding fungi, the first component (RDA1), which explained 15.65% of the total variation of fungal phyla, separated the 38-year rhizosphere and non-rhizosphere soil samples from the other samples. As shown by their close grouping and vectors, the top fungal phylum in the 38-year treatments was *Ascomycota* and was related to the AN content.

Spearman’s rank-order correlation was used to evaluate relationships between abundant phyla and soil physicochemical properties (Table S1). The results indicated different correlations of soil microbial phyla and soil physicochemical properties in the rhizosphere and non-rhizosphere soils. For example, the RA of the non-rhizosphere soil microbial phyla was negatively correlated (phylum *Firmicutes* was positively correlated) to soil pH, but there was no significant correlation with AP. In contrast, there was no significant correlation with pH and a positive correlation with AP in the rhizosphere soil.

## Discussion

In this study, Illumina MiSeq sequencing analysis of 16S rRNA and ITS sequences revealed that long-term consecutive black pepper monocropping altered the structure of the soil microbial community. The variations observed for bacteria were consistent with our previous results showing that *Acidobacteria* and *Proteobacteria* are the main phyla, with decreases in *Firmicutes*, under long-term continuous cropping^25^. In non-rhizosphere soil, the RA of *Acidobacteria* considerably increased with continuous planting, whereas *Bacteroidetes* and *Firmicutes* decreased. The variations in these three phyla were consistent with previous research on consecutive cropping^26^. With regard to fungi, *Ascomycota* was the most abundant phylum, accounting for 89.92%, and its RA increased significantly with planting duration. In rhizosphere soil, the RA of the top five phyla all increased with time. However, the *Chytridiomycota* RA decreased slightly in the rhizosphere soil, whereas the RA of the other four phyla increased. In addition, a significant decrease in the Shannon index of the rhizosphere soil was observed in the 38-year old field, suggesting that long-term consecutive cropping may cause black pepper rhizosphere soil fungal diversity to decrease significantly.

Our experimental results demonstrate that at the genus level, the abundance of the pathogenic fungus *Fusarium* increased significantly with time in both rhizosphere and non-rhizosphere soils. In contrast, *Pseudomonas* and *Bacillus*, beneficial bacteria for black pepper, showed a considerable decline. In this study, *Fusarium*, the pathogenic fungus of black pepper slow decline/wilt, significantly increased in both rhizosphere and non-rhizosphere soils. Thus, intensifying pathogen might cause black pepper to be more vulnerable to slow decline/wilt disease in long-term monoculture. Phytophthora foot rot disease and root-knot nematodes are the other two major black pepper diseases in China. *Phytophthora capsici* Leonian, which causes foot and root rot of black pepper, also referred to as “sudden wilt”, is a destructive and economically important pathogen of black pepper, occurring wherever the crop is grown^27^. *Phy. capsici* can infect all plant parts during all growth stages, and black pepper yield losses caused by *Phy. capsici* can reach 40–50%^28^. Root-knot nematodes, *Meloidogyne* spp., not only cause root damage that results in rot but also increase black pepper susceptibility to Phytophthora foot rot disease^5^. In our study, the RA of *Pseudomonas* and *Bacillus*, which have been reported to be antagonistic to *Phy. capsici*^15^ and root-knot nematode^16^, revealed a considerable decline with planting duration in both rhizosphere and non-rhizosphere soils. Increasing pathogen and decreasing antagonistic bacteria might cause black pepper to be more vulnerable to diseases in long-term monoculture fields.

In accordance with our previous study results^23^, the soil OM and AK contents and the non-rhizosphere soil pH decreased significantly but AN and AP increased with the duration of consecutive monoculture. This may have been caused by the fertilizer regime applied to the black pepper fields. In China, to obtain higher yields, growers use large amounts of N and P fertilizers, whereas organic and K fertilizers have been ignored for a long time. Applying excessive amounts of N and P fertilizers not only causes the fertilizer to be ineffective but also causes the soil pH to decrease significantly^29^,^30^. In addition, the rhizosphere soil AP content increased and was approximately 10 times higher than that in the non-rhizosphere soil; this may have been caused by root exudates, which play a major role in P bioavailability *via* several mechanisms^31^,^32^. Such conditions may enhance the activity of phosphate-solubilizing bacteria, thus increasing the plant’s P supply^33^. However, the decline in OM content in both the rhizosphere and non-rhizosphere soils suggests that more organic fertilizers should be applied in long-term consecutive monoculture of black pepper. We also found that in each black pepper field plot, the rhizosphere soil pH as well as the AK (except in the 12-year field), AN, AP and soil OM contents were all higher than in the non-rhizosphere soil. This suggests that long-term consecutive monoculture results in considerable soil nutrient unbalance and soil acidification. Although this deterioration might be a certain ease for rhizosphere soil, this would still inhibit the growth of black pepper.

The biological properties of rhizosphere soil can differ greatly from those of bulk soils^34^, and a similar phenomenon was observed in this study. First, the average RA of *Proteobacteria* in the rhizosphere soil (RA of 23.55%) was much higher than that in the non-rhizosphere soil (RA of 14.11%). Second, the RA of *Firmicutes* decreased significantly from 20.31% (12 years) to 5.37% (38 years) in the non-rhizosphere soil, but no significant difference was observed for the rhizosphere soil. Finally, at the genus level, the RA of most genera were different between the rhizosphere and non-rhizosphere soils. These results may be due to the complex interactions among root exudates, nutrient elements and soil microorganisms^35^. Moreover, soil nutrient bioavailability and microbial community richness and diversity were higher in the rhizosphere soil than in the non-rhizosphere soil. Overall, to understand the problems of consecutive monoculture of black pepper, more attention should be given to interactions among soil nutrient elements, root exudates and the microbial community, especially the function of root exudates in rhizosphere soil.

## Methods

### Sampling sites

The study was conducted at the Spice and Beverage Research Institute (18°72′N–18°76′N, 110˚19′E–110˚22′E), Wanning City, Hainan Province, P. R. China. This area is characterized by a tropical monsoon climate with an average temperature of 24.6 °C, average annual precipitation of 2,150 mm, and annual mean relative humidity of 85%.

The experimental soil samples were collected on July 15, 2015, from four different black pepper (*Piper nigrum* L. *cv.* Reyin No. 1) consecutive monoculture fields (12, 18, 28 and 38 years) with the same agronomic management and fertilization regime. For each black pepper field, nine black pepper plants were randomly selected, and soil sampled at random from each of three plants was mixed together as a single soil sample. For each plant, carefully dug up clods with black pepper roots. Soil farther than 1 cm from the roots was collected and considered as non-rhizosphere soil. Then, carefully remove the soil 5 mm away from the roots with brush and tweezers. Soil adjacent to the root segments, at 1–5 mm from the root surface, was shaken off and defined as rhizosphere soil. All soil samples were placed into sterile plastic bags, placed in an ice box, and transported to the laboratory. After passing through a 2-mm sieve, each sample was divided into two subsamples: one portion was air-dried for soil characteristic analysis, and the remainder was stored at −80 °C for DNA extraction.

### DNA extraction and Illumina MiSeq sequencing

Total DNA was extracted from 24 soil samples using a MoBio PowerSoil™ DNA Isolation Kit (Mo Bio Laboratories Inc., Carlsbad, CA, USA) according to the manufacturer’s instructions. The Genomic DNA concentration and purity were measured using a NanoDrop ND-2000 spectrophotometer (NanoDrop Technologies, Wilmington, DE).

Bacterium-specific primers 515F (GTGCCAGCMGCCGCGGTAA) and 806R (GGACTACVSGGGTATCTAAT) were used to amplify the V4 hyper-variable regions of the bacterial 16S rRNA gene. Fungus-specific primers ITS1F (CTTGGTCATTTAGAGGAAGTAA)^36^ and ITS2 (GCTGCGTTCTTCATCGATGC)^37^ were selected to target the ITS1 region. The polymerase chain reaction (PCR) was performed according to our previous publication^23^,^38^. Finally, paired-end sequencing was carried out using an Illumina MiSeq sequencer at Personal Biotechnology Co., Ltd (Shanghai, China).

### OTU-based sequence analysis

OTU-based sequence analysis was carried out using our previous methods^38^. Briefly, after removing the adaptors and primer sequences, raw sequences were assembled for each sample according to the unique barcode using QIIME^39^. Split sequences for each sample were merged using FLASH V1.2.7^40^, and low-quality sequences were discarded by QIIME. The sequences retained for each sample were processed following the established UPARSE pipeline^41^. Briefly, sequences with a quality score lower than 0.5 or a length shorter than 200 bp were removed. After discarding singletons, the remaining reads were assigned to OTUs with a threshold of 97% identity. The chimera removal processes were then performed. Finally, bacterial representative sequences were searched against the RDP database^42^,^43^, and fungal representative OTUs were classified using the UNITE database^44^.

### Determination of soil physicochemical propertiesh

Soil physicochemical properties were determined in the same manner as reported in our previous paper^23^. Briefly, soil pH was measured using a glass electrode meter in a soil-water suspension (1:5 w/v). OM and AN (hydrolyzable N) were determined using the potassium dichromate external heating method. AP was determined using the molybdenum blue method. AK was determined by flame photometry.

### Statistical analyses

Coverage, richness (Chao1 index) and diversity (Shannon index) were used to estimate the α-diversity of each sample using Mothur (version 1.36.0). Correlations between abundant soil microbial phyla and soil characteristics were determined by RDA using the vegan package of R (version 3.2.3). One-way analyses of variance (ANOVA) with Tukey’s HSD multiple range tests were performed for multiple comparisons using SPSS V20.0 (SPSS Inc., USA), and Spearman’s rank correlations were calculated using SPSS V20.0 (SPSS Inc., USA).

## Additional Information

**How to cite this article**: Li, Z. *et al*. Different responses of rhizosphere and non-rhizosphere soil microbial communities to consecutive *Piper nigrum* L. monoculture. *Sci. Rep.* **6**, 35825; doi: 10.1038/srep35825 (2016).

## Supplementary Material

Supplementary Information

## Figures and Tables

**Table 1:** Bacterial and fungal α-diversity indices in rhizosphere and non-rhizosphere soil.

Microbial community	Soil sample	Consecutive monoculturing spans	Coverage	Chao 1	Shannon
Bacteria	Rhizosphere Soil	12 y	96.49 ± 0.24 a	4930.32 ± 421.96 ab	6.72 ± 0.40 a
		18 y	96.36 ± 0.22 a	5248.75 ± 349.74 a	7.09 ± 0.16 a
		28 y	97.10 ± 1.30 a	4756.31 ± 269.36 abc	6.82 ± 0.22 a
		38 y	96.65 ± 0.21 a	4676.70 ± 376.47 abc	6.59 ± 0.26 a
	Non-rhizosphere Soil	12 y	97.33 ± 0.93 a	3854.48 ± 255.05 cd	5.92 ± 0.95 a
		18 y	96.55 ± 0.50 a	4143.82 ± 184.53 bcd	6.63 ± 0.39 a
		28 y	98.01 ± 0.93 a	3502.97 ± 482.39 d	6.22 ± 0.08 a
		38 y	97.49 ± 0.45 a	3620.26 ± 360.17 d	6.06 ± 0.34 a
Fungi	Rhizosphere Soil	12 y	97.43 ± 0.04 a	904.09 ± 23.05 a	4.46 ± 0.05 a
		18 y	97.46 ± 0.41 a	876.56 ± 31.13 a	4.20 ± 0.25 abc
		28 y	97.59 ± 0.67 a	854.45 ± 14.16 ab	4.15 ± 0.09 abc
		38 y	97.65 ± 0.04 a	822.09 ± 22.37 abc	3.88 ± 0.07 c
	Non-rhizosphere Soil	12 y	97.95 ± 0.53 a	764.67 ± 17.44 bcd	4.15 ± 0.12 abc
		18 y	97.54 ± 0.08 a	887.06 ± 3.00 a	4.29 ± 0.17 ab
		28 y	98.11 ± 0.25 a	689.43 ± 80.69 d	3.96 ± 0.12 bc
		38 y	97.98 ± 0.68 a	732.71 ± 26.32 cd	4.07 ± 0.06 bc

“12y”, “18y”, “28y” and “38y” refers to the four consecutive monoculture spans of 12, 18, 28 and 38 years, respectively. Values are presented as means ± standard deviation (n = 3). Means followed by the same letter for a given factor are not significantly different (*P *< 0.05, Turkey’s HSD test).

**Table 2:** Soil characteristics of black pepper fields.

Soil sample	Consecutive monoculturing spans	OM (g/kg)	AN (mg/kg)	AP (mg/kg)	AK (mg/kg)	pH
Rhizosphere soil	12 y	21.64 ± 0.69 a	117.48 ± 3.46 bc	204.60 ± 14.63 c	206.85 ± 13.54 b	5.90 ± 0.62 ab
	18 y	17.36 ± 0.57 b	126.93 ± 4.26 ab	342.87 ± 42.62 b	131.03 ± 9.11 cd	5.92 ± 0.20 a
	28 y	15.59 ± 0.26 c	129.85 ± 2.30 a	462.18 ± 20.24 a	168.81 ± 9.07 bc	5.83 ± 0.24 ab
	38 y	15.27 ± 0.40 c	131.03 ± 4.45 a	467.15 ± 17.87 a	141.37 ± 2.75 cd	5.35 ± 0.02 abc
Non-rhizosphere soil	12 y	14.61 ± 0.27 cd	107.96 ± 0.94 c	48.42 ± 4.01 d	253.06 ± 32.34 a	5.17 ± 0.18 bc
	18 y	12.03 ± 0.24 ef	120.44 ± 7.62 ab	49.32 ± 0.70 d	118.32 ± 3.37 d	4.71 ± 0.12 c
	28 y	13.48 ± 1.06 de	129.49 ± 3.44 a	48.20 ± 0.12 d	58.32 ± 1.41 e	4.75 ± 0.10 c
	38 y	11.91 ± 0.29 f	128.25 ± 0.98 ab	48.36 ± 0.54 d	127.53 ± 9.83 d	4.64 ± 0.01 c

“12y”, “18y”, “28y” and “38y” refers to the four consecutive monoculture spans of 12, 18, 28 and 38 years, respectively. Values are presented as means ± standard deviation (n = 3). Means followed by the same letter for a given factor are not significantly different (*P* < 0.05, Turkey’s HSD test).

## References

[b1] Sharangi A. B., Kumar R. & Sahu P. K. Survivability of black pepper (*Piper nigrum* L.) cuttings from different portions of vine and growing media. J. Crop Weed 6(1), 52–54 (2010).

[b2] Tilahun D., Tefera W. & Weldetsadik K. Macro Propagation of Black Pepper (Piper Nigrum L.). As Influenced by Growth Media and Physiological Conditions of the Stock Plant (ed. Tilahun D.) Ch. 1, 1 (LAP Lambert Academic Publishing, 2011).

[b3] Nair K. P. P. The agronomy and economy of black pepper (*Piper nigrum* L.) —The “king of spices”. Adv. Agron. 82, 271–389 (2004).

[b4] Nair K. P. P. Agronomy and Economy of Black Pepper and Cardamom (ed. Nair K. P. P.) Ch. 1, 1 (Elsevier, 2011).

[b5] Lai K. F. & Paulus A. D. Pepper Production Technology *in*Malaysia (eds Lai K. F. & Sim S. L.) Ch. 1, 1–6 (Malaysian Pepper Board, 2011).

[b6] Zu C. . Acid soil is associated with reduced yield, root growth and nutrient uptake in black pepper (*Piper nigrum* L.). Agr. Sci. 5, 466–473 (2014).

[b7] Zheng W. Q. . Chief factors influencing pepper succession cropping and prevention measures. Chinese J. Trop. Agr. 30(3), 52–55 (2010).

[b8] Yang J. F. . Status and development countermeasures of pepper industry in China. Chinese J. Trop. Agr. 30(10), 13–18 (2010).

[b9] Vargas G. S. . Field assessment of soil biological and chemical quality in response to crop management practices. World J. Microb. Biot. 25(3), 439–448 (2009).

[b10] Wu L. K. . Plant-microbe rhizosphere interactions mediated by *Rehmannia glutionosa* root exudates under consecutive monoculture. Sci. Rep.-UK 5, 15871, doi: 10.1038/srep15871 (2015). PMC4626807 26515244

[b11] He J. Z., Zheng Y. & Chen C. R. Microbial composition and diversity of an upland red soil under long-term fertilization treatments as revealed by culture-dependent and culture-independent approaches. J. Soil Sediment. 8, 349–358 (2010).

[b12] Yu H., ZU C., WU H. S., Yang J. F. & Zheng W. Q. The difference analysis of quality in pepper from different planting years. Chinese J. Trop. Crop 34(5), 850–854 (2013).

[b13] LI Z. G. . Soil physicochemical properties and microbial dynamics in the fields with the different aging black pepper. Chinese J. Trop. Crop 33(7), 1245–1249 (2012).

[b14] Yang J. F. . Effects of the planting period on the soil chemical fertility parameters in pepper garden. Chinese J. Trop. Crop 32(4), 592–597 (2011).

[b15] Tran H. & Kruijt M. J. Diversity and activity of biosurfactant-producing *Pseudomonas* in the rhizosphere of black pepper in Vietnam. J. Appl. Microbiol. 104(3), 839–851 (2008). 10.1111/j.1365-2672.2007.03618.x 17976176

[b16] Devapriyanga R., Jonathan E. I., Meena K. S. & Kavitha P. G. Bioefficacy of *Pseudomonas* and *Bacillus* isolates against root-knot nematode, *Meloidogyne incognita* in black pepper cv. Panniyur 1. *Indian* J. Nematol. 42(1), 57–62 (2012).

[b17] Raaijmakers J. M., Paulitz T. C., Steinberg C., Alabouvette C. & Moënne-Loccoz Y. The rhizosphere: a playground and battlefield for soilborne pathogens and beneficial microorganisms. Plant Soil 321, 341–361 (2009).

[b18] Berendsen R., Pieterse C. & Bakker P. The rhizosphere microbiome and plant health. Trends Plant Sci. 17, 478–486 (2012). 22564542 10.1016/j.tplants.2012.04.001

[b19] Klemedtsson L., Svensson B. H. & Rosswall T. Relationships between soil moisture content and nitrous oxide production during nitrification and denitrification. Biol. Fert. Soils 6(2), 106–111 (1988).

[b20] Mukerji K. G., Manoharachary C. & Singh J. Microbial Activity in the Rhizosphere (eds Mukerji K. G. .). (Springer publishing, 2006).

[b21] Zhao Y. Z. . Interaction of *Pseudostellaria heterophylla* with *Fusarium oxysporum* f. sp. *heterophylla* mediated by its root exudates in a consecutive monoculture system. Sci. Rep.-UK 5, 8197, doi: 10.1038/srep08197 (2015). PMC4314652 25645742

[b22] Jakub E. & Jaroslav Z. The comparison of microbial activity in rhizosphere and non-rhizosphere soil stressed by drought. Mental. Net. 234–240 (2014).

[b23] Sari S., Anu E. & Minna K. M. Regulation of microbial community composition and activity by soil nutrient availability, soil pH, and herbivory in the Tundra. Ecosystems 15, 18–33 (2012).

[b24] Jennifer K. C., Deirdre R., Deirdre B. G. & Nicholas C. Altering the mineral composition of soil causes a shift in microbial community structure. Fems, Microbiol. Ecol. 61(3), 414–423 (2007). 17681010 10.1111/j.1574-6941.2007.00361.x

[b25] Xiong W. . The effect of long-term continuous cropping of black pepper on soil bacterial communities as determined by 454 pyrosequencing. PLoS ONE 10(8), e0136946, doi: 10.1371/journal.Pone. 0136946 (2015). 26317364 PMC4552827

[b26] Lauber C. L., Hamady M., Knight R. & Fierer N. Pyrosequencing-based assessment of soil pH as a predictor of soil bacterial community structure at the continental scale. Appl. Environ. Microbiol. 75, 5111–5120 (2009). 19502440 10.1128/AEM.00335-09 PMC2725504

[b27] George C. K., Abdullah A. & Chapman K. Pepper production guide for Asia and the Pacific. Joint effort of theInternational Pepper Community (IPC) and Food and Agricultural Organization of United Nations (FAO). Bangkok, Thailand:IPC (2005).

[b28] Drenth A. & Sendall B. Economic impact of Phytophthora disease in Southeast Asia. Diversity and Management of Phytophthora in Southeast Asia (eds. Drenth A. & Guest D. I.) Ch. 2, 10–28 (Australian Centre for International Agricultural Research, 2004).

[b29] Malhi S. J., Nyborg M. & Harapiak J. T. Effects of long term N fertilizer induced acidification and liming on micronutrients in soil land in brome grass hay. Soil Till. Res. 48, 91–101 (1998).

[b30] Jackie L. S. . Soil acidification from long-term use of nitrogen fertilizers on winter wheat. Soil Sci. Soc. Am. J. 75, 957–964 (2011).

[b31] Hinsinger P. Bioavailability of soil inorganic P in the rhizosphereas affected by root-induced chemical changes: a review. Plant Soil 237, 173–195 (2001).

[b32] Vance C. P., Uhde-Stone C. & Allan D. L. Phosphorus acquisition and use: critical adaptations by plants for securing a nonrenewable resource. New Phytol. 157, 423–447 (2003). 10.1046/j.1469-8137.2003.00695.x 33873400

[b33] Richardson A. E. Prospects for using soil microorganisms to improve the acquisition of phosphorus by plants. Aust. J. Plant Physiol. 28, 897–906 (2001).

[b34] Hinsinger P., Bengough A. G., Vetterlein D. & Young I. M. Rhizosphere: biophysics, biogeochemistry and ecological relevance. Plant Soil 321, 117–152 (2009).

[b35] Jason A. P. . Diversity and heritability of the maize rhizosphere microbiome under field conditions. PNAS 110(16), 6548–6553 (2013). 23576752 10.1073/pnas.1302837110 PMC3631645

[b36] Gardes M. & Bruns T. D. ITS primers with enhanced specificity for *basidiomycetes*-application to the identification of mycorrhizae and rusts. Mol. Ecol. 2, 113–118, doi: 10.1111/j.1365-294X.1993. tb00005. x (1993). 8180733

[b37] White T. J., Bruns T., Lee S. & Taylor J. W. Amplification and direct sequencing of fungal ribosomal RNA genes for phylogenetics. PCR Protoc. Guide Methods Appl. 18, 315–322, doi: 10.1016/b978-0-12-372180-8.50042-1 (1990).

[b38] Xiong W. . Comparison of fungal community in black pepper-vanilla and vanilla monoculture systems associated with vanilla *Fusarium* Wilt Disease. Front. Microbiol. 7, 117, doi: 10.3389/fmicb.2016. 00117 (2016). 26903995 PMC4746283

[b39] Caporaso J. G. . QIIME allows analysis of high-through put community sequencing data. Nat. Methods 7, 335–336 (2010). 20383131 10.1038/nmeth.f.303 PMC3156573

[b40] Magoč T. & Salzberg S. L. FLASH: fast length adjustment of short reads to improve genome assemblies. Bioinformatics 27, 2957–2963, doi: 10.1093/bioinformatics/btr507 (2011). 21903629 PMC3198573

[b41] Edgar R. C. UPARSE: highly accurate OTU sequences from microbial amplicon reads. Nat. Methods 10, 996–998, doi: 10.1038/nmeth.2604 (2013). 23955772

[b42] Kõljalg U. . Towards a unified paradigm for sequence-based identification of fungi. Mol. Ecol. 22, 5271–5277, doi: 10.1111/mec.12481 (2013). 24112409

[b43] Cole J. R. . The ribosomal database project: improved alignments and new tools for rRNA analysis. Nucl. Acids Res. 37, 141–145 (2009). 10.1093/nar/gkn879 PMC2686447 19004872

[b44] Wang Q., Garrity G. M., Tiedje J. M. & Cole J. R. Naive bayesian classifier for rapid assignment of rRNA sequences into the new bacterial taxonomy. Appl. Environ. Microbiol. 73, 5261–5267 (2007). 17586664 10.1128/AEM.00062-07 PMC1950982

