# Supplementary material for: Different responses of rhizosphere and non-rhizosphere soil microbial communities to consecutive Piper nigrum L. monoculture
Source: Sci Rep. 2016 Oct 24;6:35825. doi: 10.1038/srep35825 (PMC5075767; doi:10.1038/srep35825)
Supplement: Supplementary Information [file srep35825-s1.pdf]

Different responses of rhizosphere and non-rhizosphere soil microbial communities to consecutive *Piper nigrum* L. monoculture

Zhigang Li[1], Chao Zu[1], Can Wang[1], Jianfeng Yang[1], Huan Yu[1] & Huasong Wu[1]*

1. Spice and Beverage Research Institute, Chinese Academy of Tropical Agricultural Sciences & Key Laboratory of Genetic Resources Utilization of Spice and Beverage Crops, Ministry of Agriculture & Hainan Provincial Key Laboratory of Genetic Improvement and Quality Regulation for Tropical Spice and Beverage Crops, Wanning 571533, Hainan, P. R. China.

***Correspondence author, H. S. W. (email: 13807622912@163.com)**

Supplementary Information

Supplementary Figure S1. Overall bacterial (a) and fungal (b) communities in the 24 soil samples from four consecutive monoculture of black pepper fields. Average relative abundances (ARAs) were calculated as the proportion of individual bacterial and fungal taxa to the total number of 16S rRNA or ITS gene sequence reads.

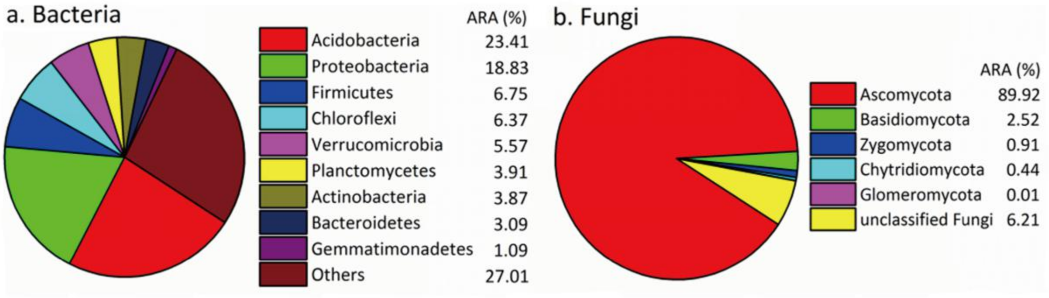

Supplementary Table S1. Spearman's rank correlation (r) between abundant taxa and soil properties.

			OM	AN	AP	AK	pH
Rhizosphere soil	Bacteria	*Acidobacteria*	0.673*	-0.694*	-0.563	0.816**	0.107
		Proteobacteria	-0.636*	0.660*	0.622*	-0.413	0.320
		Firmicutes	0.417	-0.482	-0.353	0.643*	0.343
		Chloroflexi	-0.140	0.005	0.137	-0.047	0.390
		Verrucomicrobia	-0.570	0.400	0.546	-0.694*	-0.450
		Actinobacteria	-0.884**	0.813**	0.859**	-0.679*	-0.470
		Planctomycetes	-0.149	0.218	0.041	-0.531	-0.274
		Bacteroidetes	-0.302	0.175	0.212	-0.617*	-0.112
		Gemmatimonadetes	-0.618*	0.585*	0.676*	-0.187	0.016
		Nitrospira	0.631*	-0.643*	-0.507	0.725**	-0.077
	Fungi	*Ascomycota*	-0.774**	0.675*	0.837**	-0.286	-0.489
		Basidiomycota	-0.098	0.317	-0.053	-0.540	-0.034
		Chytridiomycota	0.044	0.021	-0.005	0.342	0.310
		Glomeromycota	0.459	-0.448	-0.534	0.291	-0.142
		Zygomycota	-0.132	0.140	-0.005	-0.599*	0.255
Non-rhizosphere soil	Bacteria	*Acidobacteria*	-0.713**	0.743**	-0.056	-0.717*	-0.843**
		Proteobacteria	-0.575	0.535	-0.518	-0.485	-0.397
		Firmicutes	0.777*	-0.783**	-0.091	0.877**	0.890**
		Chloroflexi	0.494	0.086	-0.032	-0.328	0.178
		Verrucomicrobia	-0.472	0.374	0.302	-0.465	-0.548
		Actinobacteria	-0.885**	0.534	0.212	-0.408	-0.695*
		Planctomycetes	-0.647*	0.666*	0.237	-0.608*	-0.815**
		Bacteroidetes	-0.382	-0.265	0.257	0.095	-0.070
		Gemmatimonadetes	-0.134	0.473	0.133	-0.669*	-0.623*
		Nitrospira	0.246	-0.385	-0.140	0.522	0.524
	Fungi	*Ascomycota*	-0.588	0.570	-0.016	-0.269	-0.597*
		Basidiomycota	0.463	0.231	-0.074	-0.385	-0.136
		Chytridiomycota	0.372	-0.227	0.122	0.068	0.460
		Glomeromycota	0.457	0.194	0.061	-0.279	-0.240
		Zygomycota	-0.137	-0.197	-0.157	-0.095	-0.060

*P<0.05; **P<0.01
